# Incidence and prevalence of acquired brain injury in children and young people globally: A systematic review and meta-analysis protocol

**DOI:** 10.1371/journal.pone.0357762

**Published:** 2026-10-08

**Authors:** Sophie Orchard, Kate Radford, Joseph C. Manning, Rachel Keetley, Laila J. Tata

**Affiliations:** 1 Lifespan and Population Health, School of Medicine, University of Nottingham, Nottingham, United Kingdom; 2 Nottingham NIHR Biomedical Research Centre, University of Nottingham, Nottingham, United Kingdom; 3 NIHR Applied Research Collaboration East Midlands, University of Nottingham, Nottingham, United Kingdom; 4 Centre for Rehabilitation & Ageing Research, University of Nottingham, Nottingham, United Kingdom; 5 Division of Nursing Science, Pregnancy and Child Health, School of Healthcare, College of Life Sciences, University of Leicester, Leicestershire, United Kingdom; 6 Nottingham Children’s Hospital, Family Health Clinical Care Group, Nottingham University Hospitals NHS Trust, Nottingham, United Kingdom; 7 Nottingham Children’s Hospital, Nottingham University Hospitals NHS Trust, Nottingham, United Kingdom; 8 Centre for Perinatal Research, University of Nottingham, Nottingham, United Kingdom; University of Florida, UNITED STATES OF AMERICA

## Abstract

**Introduction:**

Acquired brain injury refers to any injury or damage to the brain post-birth. It is considered a silent epidemic due to late or missed diagnosis, despite continued interaction with healthcare and longer-term impacts. There is a paucity of robust contemporary evidence that quantifies the population burden of childhood acquired brain injuries. This study aims to estimate the global burden of childhood acquired brain injury by pooling estimates of incidence and prevalence overall, by subtype, and by severity. We will assess variation by time, sociodemographic and clinical characteristics, and geography.

**Methods & analyses:**

MEDLINE, EMBASE, CINAHL, and Scopus publication databases will be searched. Eligible studies will have acquired brain injury incidence or prevalence data on 0–18-year-old individuals within a defined general population-based group from the year 2000 onward. Study design, acquired brain injury and baseline characteristics will be extracted, and quality will be assessed using the JBI Critical Appraisal Checklist. A random effects meta-analysis will be conducted to pool incidence and prevalence. To assess heterogeneity where there is sufficient data availability, subgroup meta-analyses and a meta-regression will be performed by time, age, sex, ethnicity, health/clinical factors, preexisting conditions, socioeconomic factors, country or geographic region.

**Ethics & dissemination:**

No formal ethics approval is needed due to no primary data collection. Dissemination will be through peer-reviewed publication and contribution to a PhD thesis developing the utility of routinely collected healthcare data to study acquired brain injury. Results will quantify the burden of childhood acquired brain injury by providing a comprehensive synthesis of global data, identifying geographical disparities and data gaps. This review will be beneficial to healthcare professionals and policymakers. Registration with PROSPERO International Prospective Register of Systematic Reviews (CRD420250654936).

## Introduction

Acquired brain injury (ABI) affects every population group with no limit on location or socioeconomic status [1,2]. It is an umbrella term for both traumatic and nontraumatic injuries to the brain occurring after birth [2–5]. A traumatic brain injury (TBI) is caused by a hit, jolt, or penetration externally to the head [3,4], whilst a non-traumatic brain injury is caused by events internal to the body, such as stroke, tumour, infection, or inflammation [3]. Damage from a brain injury typically occurs immediately following the causal event, however it is not always recognised or detected immediately. Damage may be detected up to years later through physical, social, educational and psychological functioning [6–10], which can have sustained and wide reaching impacts on the lives of the individuals and their families [4,9].

ABI has been reported as a silent epidemic due to the lack of research focus, including limited incidence and prevalence estimations and recognised underdiagnosis or late diagnosis, especially for children and young people [1–6,9,11–13]. A recent scoping review of current support needs concluded that children and young people affected by acquired brain injury, as well as their families and those involved in their care, have extensive needs post-injury; specifically in terms of rehabilitation and long-term support [14]. Planning services to address the extent and types of specific needs is difficult due to variations in how ABI incidence and prevalence is captured and which ABI groups are studied [1].

A comprehensive literature review including incidence rates of paediatric TBI using studies from all over the world between 1980 and 2010 reported that estimates ranged from 12 (Sweden) to 486 (Australia) per 100,000 children and young people each year [2]; no meta-analysis was conducted. Whilst these estimates provide valuable insight of TBI in specific geographical regions, the temporal scope of the data collection in this review [2]does capture contemporary data, important for informing current services.

Another study from the United States of America, published in 2021, estimated a much higher incidence of childhood TBI, 1.2% paediatric TBI cases per year using a 12 year population-based survey [15]. It has also been reported that occurrence varies considerably by age; for example in Korea it has been reported that 0–4-year-olds have the highest prevalence of TBI [16], whereas this peak is seen in 6–10-year olds in Kashmir [17]. These estimates expose this condition’s shifting vulnerability across the ages globally.

Whilst TBI is recognised as a leading cause of acquired brain injury, it is important to note that other subtypes of acquired brain injury also contribute to the incidence and prevalence of this condition, such as stroke, encephalitis, and brain tumours. However, there is a notable discrepancy in the amount of literature available focused on these subtypes.

Stroke remains one of the most prevalent causes of global death and disability [18,19]. It has been estimated that in 2019 the global incidence of paediatric stroke was 18 cases per 100,000 children, with ischemic stroke being more common in children under the age of 5 and haemorrhagic stroke more common in adolescents [19].

Brain tumour related research has estimated a mortality rate of 0.66 per 100,000 children aged 0–14 years old in the United States of America [20]. A systematic analysis of the Global Burden of Disease Study estimated a declining annual mortality rate from 1.31 per 100,000 children in 1990 to 0.91 in 2019 [11]. It remains unclear if this decline was due to decreased incidence or improved survival and raises uncertainty surrounding the lifelong burden of tumour related brain injuries.

Encephalitis related brain injury poses another significant cause of ABI burden yet remains underrepresented in the literature. Whilst a higher risk of encephalitis is noted in children in comparison to adults [13], paediatric-specific estimates are lacking.

Since existing research mainly describes subtypes of acquired brain injury, there remains a lack of understanding of the overall burden of acquired brain injury. To effectively guide prevention strategies, intervention programmes, and healthcare planning for the short- and long-term needs following ABI, it is essential to quantify the overall burden of paediatric ABI alongside its subtypes. This will not only support healthcare resource allocation but also highlight the critical needs within educational services and broader social care systems.

This research will summarise the evidence on the incidence and prevalence of ABI among 0–18-year-olds and highlight evidence gaps by conducting a systematic review to estimate the occurrence of ABI overall and by its subtypes and severity. The review also aims to describe how these vary by individual and sociodemographic factors, geographic region and changes over time.

## Methods

### Study registration

This study has been registered in the PROSPERO International Prospective Register of Systematic Reviews (CRD420250654936). This register will be updated regarding the study timeline as the review progresses. The proposed completion date is currently December 2026. This protocol was developed using the Preferred Reporting Items for Systematic Review Protocols (PSIMA-P) guidelines [21] and follows the Preferred Reporting Items for Systematic Reviews Search (PRISMA-S) [22] (see S1 Appendix for the completed PRISMA-P checklist and S2 Appendix for the PRISMA-S checklist).

Studies will be screened and selected according to the criteria outlined below.

### Eligibility criteria

#### Population and time period.

The population for this review will be children and young people aged between 0 and 18 years old from any country globally with data from the year 2000 onwards. The source must be general population-based in that the sample enables calculation of population-based incidence or prevalence. Studies with data on people over age 18 will only be included if it is possible to separately extract data on those age 0–18. Studies with data before 2000 will only be included if it is possible to separately extract post-2000 data.

#### Outcomes.

The primary outcome of this review will be ABI in 0–18-year-olds, measured as incidence (ABI newly diagnosed or newly identified as a proportion of people in a defined population over a specified time period) or prevalence (proportion of a defined population identified as having ABI at a particular point or period in time). Studies will be eligible if they report the incidence and/or prevalence of a predefined ABI diagnosis. Studies reporting only chronic neurological sequelae, functional impairments, rehabilitation needs, or secondary injury processes without description of the underlying ABI diagnosis will be excluded. Secondary injury processes will be considered indicators of injury severity or clinical characteristics rather than separate ABI outcomes. For this review, acquired brain injury (ABI) is defined as an injury to the brain occurring after birth that results from traumatic, non-congenital, or non-degenerative pathological processes. Eligible ABI subtypes include: (1) traumatic brain injury (TBI); (2) stroke, including ischaemic and haemorrhagic stroke; (3) encephalitis; and (4) brain tumour-related brain injury. ABI may be identified in any setting, including primary care, specialist healthcare, hospital, community or educational services.

The review will exclude congenital or genetic conditions affecting brain development during or after gestation, neurodevelopmental disorders, progressive neurodegenerative diseases, and brain injuries occurring during the perinatal period or at birth, including birth trauma and complications of prematurity. Studies examining other causes of acquired brain injury will be considered only where the diagnosis can be clearly classified within the predefined ABI subtype or reported separately.

#### Study design.

Studies reporting a population-based incidence and/or prevalence of ABI will be considered for this review. Whilst this will primarily include cohort and cross-sectional studies, any observational studies that include a study population representative of the general population in a defined geographic area will be eligible for inclusion, e.g., a city, region or country. Hospital-based studies from a single centre or a network of hospitals will only be included if they describe how their admissions are from a particular population base or catchment area and are thus representative of the general population in a defined geographic area, reporting the relevant baseline population. No language restrictions will be used.

#### Information sources and search strategy.

We will search four electronic databases (MEDLINE and EMBASE via Ovid, CINAHL via EBSCO, and Scopus) and restrict the search for studies published from 2000 onwards. The year 2000 cutoff was chosen to ensure relevance to current clinical settings and because of limited digital data capture on a wide population scale before the year 2000.

Grey literature sources will also be searched, including ProQuest Dissertations & Theses, the Kings Fund (via The Health Management Information Consortium (HMIC) database via Ovid), and MedRxiv. Reference lists of all included studies will also be searched.

Search strategies have been developed using medical subject headings (MeSH) and free text searches related to ABI, its subtypes, population demographics, study design and time restrictions. These terms will be translated into relevant terms for each database interface that does not use MeSH terms. A workshop with a brain injury clinical and academic expert stakeholder group was held to identify any relevant MeSH terms and free-text search strings relevant to this review which was used to develop the initial MEDLINE search strategy (S3 Appendix). This the search strategy will be translated into each database syntax and subject heading style.

### Study records

#### Data management.

Literature search outputs will be uploaded to Covidence Systematic Review Software, a web-based tool that facilitates collaboration amongst reviewers during the screening process. We will use this software to identify and remove any duplicates from the screening total and to conduct screening for study inclusion.

#### Selection process.

Following de-duplication, dual screening will be used for study inclusion in two stages. For stage 1, title and abstract screening, SO will screen all studies, and a team of secondary reviewers will each screen 20% of all studies. To proceed to the full text screening stage, a study should meet the following criteria: 1) report an incidence and/or prevalence of ABI overall or at least one of the specified ABI diagnoses (traumatic brain injury, stroke, encephalitis, or brain tumour) or data to calculate these, 2) the study aimed to obtain a representative sample of children with ABI whose injuries occurred between 0–18 years old, and 3) the children in the baseline represent a general population-based study sample or externally available relevant general-population data are used with a clearly referenced source. Any uncertainty or disagreement between reviewers will be discussed to resolve screening decisions, however, if agreement cannot be met on these criteria, a third reviewer will independently screen the study and make a decision; the study will proceed to full text for review of key details if any uncertainty remains. For stage 2, all studies reaching the full text screening stage will all be dual screened by the reviewing team. To be included in the final review and proceed to data extraction the following will be checked to ensure they fully meet the inclusion criteria: 1) if any incidence/prevalence data is collected pre-2000, the 2000-onwards data is separately extractable, 2) if any incidence/prevalence data is collected on a population without age restriction, that any data for children between age 0–18 is separately extractable, and 3) each study must report the number of children and young people with ABI overall or at least one of the ABI diagnoses, and report the number of children in the denominator used for incidence and/or prevalence calculations (if one or both of these details are missing, the author will be contacted for these details). If any conflicts arise at this stage, reviewers will meet to discuss the conflicts and review the paper together; if agreement cannot be met based on the inclusion criteria, a third reviewer will be sought to make the decision.

A PRIMSA flow diagram will be created and used to summarise this process.

#### Data collection process.

For all studies included after screening, data will be extracted using a bespoke data extraction form developed in Microsoft Excel. Dual data extraction will be conducted with SO extracting data from all studies and independent extraction from the team of secondary reviewers each assigned a subset of the identified studies for extraction. Any discrepancies will be settled through discussion and consultation with a third team member if consensus cannot be reached.

#### Data items.

The following data items will be extracted into the bespoke form: publication data (article type, journal of publication, author list, title, year of publication, DOI, patient and public involvement, ethical considerations, conflicts of interest, and study approval), general study details (aims and/or objectives, study design, data type and source, geographic representation of the study, and the time period studied), population data (age, sex, inclusion and exclusion criteria of the study population; the proportion of children and young people without an ABI that was used as the denominator measure of incidence and prevalence; the proportion of children and young people with a relevant ABI; finally any incidence and/or prevalence estimates of ABI, its subtypes, and/or individual level or area-level factors including age, ethnicity, health/clinical factors and/or preexisting conditions, sociodemographic characteristics, country or region, or time period, and their confidence intervals), and outcomes (the type of ABI; how the ABI is ascertained, whether this be a clinically confirmed diagnosis, self-reported, or within education, community, or population-based settings; how the severity was measured; and any reported mechanisms of injury). Where reported, secondary injury processes will be extracted as clinical characteristics or measures of injury severity. If any of the key data items from the population data section were missing from a study, authors were contacted to obtain the information.

#### Risk of bias in individual studies.

The JBI tool “Critical Appraisal Checklist for Prevalence Studies’ will be used by each reviewer at the data extraction stage to assess the risk of bias of each included study [23]. This checklist assesses the quality of sampling and population representation, the quality of the data and analysis, and the response and bias management of each study. Any disagreements between reviewers will be settled via discussion; if agreement cannot be reached an additional reviewer will be consulted.

#### Data synthesis

A narrative synthesis of all studies based on the data extracted will be provided which will be described both in the text and tabulated. The number of children and young people with and without an ABI or ABI subtype from each study will be used to calculate pooled estimates of the incidence (ABI newly diagnosed or newly identified as a proportion of people in a defined population over a specified time period) and/or prevalence (proportion of a defined population identified as having ABI at a particular point or period in time) of ABI with 95% confidence intervals across all included studies using a random-effects meta-analysis in the statistical software R.

Meta-analyses will only be conducted on studies reporting statistics or clinically relevant details where we deem them to have the same or similar injury aetiology (subtype) and outcome measures. Unless there are many representative studies across different ABI aetiologies, studies will not be pooled across all ABI aetiologies due to the potential heterogeneity limiting clinically useful interpretation. Where substantial methodological heterogeneity exists, findings will be synthesised narratively without meta-analysis following the SWIM guidelines [24]. To conduct the meta-analysis a minimum of 2 studies will be required. The I^2^ statistic will be used to quantify heterogeneity. Where sufficient data are available, subgroup analyses and meta-regression will be undertaken according to ABI ascertainment method and data source [25]. Studies will only be pooled where case definitions and data collection methods are considered sufficiently comparable. This may include incidence and prevalence of ABI by sub-group factors including age, ethnicity, injury severity, sociodemographic measures, health/clinical factors or preexisting conditions, geography, and quality measures relating to ABI ascertainment such as clinically confirmed versus self-reported ABI. As studies will likely use different age cut-offs or bands when reporting their data, we will predefine our age brackets according to developmental milestones which impacts differences in incidence of certain injury mechanisms: Up to 1 year old (infancy), 1–4 years, 5–9 years, 10–14 years, and 15–18 years. Studes will be pooled for analysis where the data reported meets the age brackets set out; if they do not meet this, they will be narratively described. We will use a similar approach to decide logical categories for other sub-group factors based on the available data.

There are limited validated tools that measure ABI severity, and available tools are not consistently used in clinical practice. As such, there is inconsistency in reporting or no reporting of ABI severity in research studies. Considering this, we will only combine and pool estimates across studies where the severity metrics used are comparable, and where not appropriate we will narratively describe the incidence and prevalence. To evaluate any publication bias, Egger’s test and a funnel plot will be performed to determine the significance of any asymmetry; the significant p-value threshold will be 0.05.

## Discussion

This systematic review and meta-analysis will provide the first comprehensive global summary and pooled estimates of the incidence and prevalence of ABI or ABI subtypes in children and young people aged 0–18 years, synthesising existing epidemiological literature across diverse populations and settings. This review will not include single centre studies unless they demonstrate to be representative of a defined geographical population base. Whilst this may exclude some clinically valuable ABI studies, the purpose of this review is to summarise the available evidence that specifically describes a population-based risk. By aiming to describe and disaggregate data by injury subtype, severity, age, sex, ethnicity, pre-existing health conditions, sociodemographic factors, and geographical regions, this study will offer a nuanced understanding of the distribution and determinants of ABI worldwide. It will highlight the ABI data gaps according to subtypes, groups and geographies to inform the need for future research or surveillance.

Previous research has confirmed the lack of current services for follow-up and/or rehabilitation [14], providing example areas of need such as the physical, psychological, cognitive, and social impairments experienced as a consequence of ABI. Quantifying the global burden of ABI in this population is critical for allowing service providers to understand this condition [26] and is vital for providing quantitative estimates of resource requirements [12], and the necessary scale of support that is needed to meet the needs of patients and families with ABI experience [14,27]. The findings from this review will illuminate under-researched areas and methodological gaps, thereby guiding future research priorities and service development.

This work will contribute to a more coherent and actionable evidence base to support children and young people affected by ABI, particularly in low-resource settings where data are often sparse or inconsistent.

The review will be conducted in accordance with established methodological standards, ensuring transparency, reproducibility, and analytical rigour. Upon completion, the findings will be disseminated through peer-reviewed publication and incorporated into a doctoral thesis, with the aim of influencing both academic discourse and practical frameworks for care and rehabilitation.

## Supporting information

S1 Appendix PRISMA-P checklist. (DOCX)

S2 Appendix PRISMA-S checklist for protocols. (DOCX)

S3 Appendix MEDLINE search strategy. (DOCX)
