# Supplementary material for: Incidence and prevalence of acquired brain injury in children and young people globally: A systematic review and meta-analysis protocol
Source: PLoS One. 2026 Oct 8;21(10):e0357762. doi: 10.1371/journal.pone.0357762 (PMC13648995; doi:10.1371/journal.pone.0357762)
Supplement: Supplementary file 3 [file pone.0357762.s003.docx]

# S3 Appendix: MEDLINE search strategy

Draft MEDLINE search – OVID interface s3

1. exp Brain Injuries, Diffuse/
2. exp Brain Injuries, Traumatic/
3. exp Brain Injury, Chronic/
4. exp Intracranial Hemorrhages/
5. exp Brain Neoplasms/
6. exp Encephalitis/
7. exp Meningitis/
8. exp Stroke/
9. exp Brain Hemorrhage, Traumatic/
10. ABI.ti,ab,kf
11. TBI.ti,ab,kf
12. encephalit*.ti,ab,kf.
13. meningit*.ti,ab,kf.
14. (head adj3 (injur* or trauma*)).ti,ab,kf.
15. ((brain or cerebr*) adj3 (injur* or trauma* or h?emorrhag*)).ti,ab,kf.
16. (stroke* or "brain bleed*").ti,ab,kf.
17. (brain adj3 (tumo?r* or cancer* or metastas* or neoplasm*)).ti,ab,kf.
18. (brain adj3 (inflam* or swell*)).ti,ab,kf.
19. (brain adj3 infect*).ti,ab,kf.
20. 1 or 2 or 3 or 4 or 5 or 6 or 7 or 8 or 9 or 10 or 11 or 12 or 13 or 14 or 15 or 16 or 17 or 18 or 19
21. 20 not (tubercul* or TB).ti,ab,kf.
22. exp adolescent/ or exp child/ or exp infant/
23. (adolescen* or babies or baby or child* or infan* or kid? or neonat* or neo-nat* or newborn* or new-born* or paediatric* or peadiatric* or pediatric* or teen* or toddler? or youth*).ti,ab,kf.
24. 22 or 23
25. exp Incidence/ or exp prevalence/
26. (incidence or prevalence).ti.
27. 25 or 26
28. exp Epidemiologic studies/
29. exp cohort studies/
30. exp Cross-Sectional Studies/
31. (cross sectional adj3 study).ti,ab,kf.
32. (population adj3 study).ti,ab.
33. (longitudinal adj3 study).ti,ab.
34. (cohort adj study).ti,ab,kf.
35. 28 or 29 or 30 or 31 or 32 or 33 or 34
36. 21 and 24 and 27 and 35
37. limit 36 to (humans and yr="2000 -Current")
