# Supplementary material for: Incidence and prevalence of acquired brain injury in children and young people globally: A systematic review and meta-analysis protocol
Source: PLoS One. 2026 Oct 8;21(10):e0357762. doi: 10.1371/journal.pone.0357762 (PMC13648995; doi:10.1371/journal.pone.0357762)
Supplement: Supplementary file 4 [file pone.0357762.s005.docx]

July 30, 2026

Dear PLoS One editor and reviewers,

We would like to thank both the editor and the reviewers for taking the time to read and review our manuscript, and for providing constructive feedback. We have revised the manuscript in response to your comments and have provided a point-by-point response below.

For ease, we have italicised the reviewers’ comments, and our author responses follow in plain text; we have also added page number and line numbers within responses where appropriate that relate to the revised manuscript.

Reviewer 1 comment 1:

*“This is a well-structured and timely protocol addressing an important clinical and public health gap. The manuscript clearly articulates the rationale for a global synthesis of childhood acquired brain injury epidemiology, and the methodological approach is rigorous and appropriate for a systematic review and meta-analysis.*

*The protocol is comprehensive in scope, incorporating both traumatic and non-traumatic brain injuries across multiple subtypes (TBI, stroke, tumors, encephalitis). The methods are detailed and transparent, with appropriate use of established frameworks (PRISMA-P and PRISMA-S guidelines, JBI Critical Appraisal Checklist). The planned subgroup analyses by age, geography, ethnicity, and socioeconomic factors will provide valuable granularity for identifying disparities and service gaps. Registration with PROSPERO and the inclusion of grey literature strengthen reproducibility and reduce bias. The clinical significance is clear, as quantifying the global burden will meaningfully inform service planning and policy.”*

Author reply to reviewer 1 comment 1:

We thank the reviewer for the positive comments surrounding the relevance of our protocol. We agree and thank them also for their recollection of the robust methodology presented and have retained this in the revised manuscript.

Reviewer 1 comment 2:

*“In the Study Records/Selection Process section, it is stated that conflicts between reviewers will be resolved through discussion or consultation with a third team member. Consider briefly specifying a priori decision rules or criteria that will guide these discussions (for example, how borderline cases at the inclusion/exclusion threshold will be handled, or whether specific study characteristics will trigger mandatory third-reviewer consultation). This adds an additional layer of transparency and helps prevent post-hoc decision-making during screening.”*

Author reply to reviewer 1 comment 2:

Thank you for this suggestion. We have now added on page 10-11 lines 226-270 a description of how a third reviewer may be used, this is as follows: “Following de-duplication, dual screening will be used for study inclusion in two stages. For stage 1, title and abstract screening, SO will screen all studies, and a team of secondary reviewers will each screen 20% of all studies. To proceed to the full text screening stage, a study should meet the following criteria: 1) report an incidence and/or prevalence of ABI overall or at least one of the specified ABI diagnoses (traumatic brain injury, stroke, encephalitis, or brain tumour) or data to calculate these, 2) the study aimed to obtain a representative sample of children with ABI whose injuries occurred between 0-18 years old, and 3) the children in the baseline represent a general population-based study sample or externally available relevant general-population data are used with a clearly referenced source. Any uncertainty or disagreement between reviewers arises will be discussed to resolve screening decisions, however, if agreement cannot be met on these criteria, a third reviewer will independently screen the study and make a decision; the study will proceed to full text for review of key details if any uncertainty remains. For stage 2, all studies reaching the full text screening stage will all be dual screened by the reviewing team. To be included in the final review and proceed to data extraction the following will be checked to ensure they fully meet the inclusion criteria: 1) if any incidence/prevalence data is collected pre-2000, the 2000-onwards data is separately extractable, 2) if any incidence/prevalence data is collected on a population without age restriction, that any data for children between age 0-18 is separately extractable, and 3) each study must report the number of children and young people with ABI overall or at least one of the ABI diagnoses, and report the number of children in the denominator used for incidence and/or prevalence calculations (if one or both of these details are missing, the author will be contacted for these details). If any conflicts arise at this stage, reviewers will meet to discuss the conflicts and review the paper together; if agreement cannot be met based on the inclusion criteria, a third reviewer will be sought to make the decision..”

Reviewer 2 comment 1:

*“This systematic review and meta-analysis addresses an important and understudied topic. The focus on population-based data and contemporary estimates has the potential to provide valuable information for healthcare and service planning as well as policy for children with ABI. The manuscript is clearly written, the age range is well specified, and there is a thoughtful attempt to consider ABI across subtypes and severities. With some refinement of the scope and clearer operational definitions, this protocol and eventual study could make a substantial contribution to pediatric ABI literature. Below are specific comments and suggestions.”*

Author reply to reviewer 2 comment 1:

Thank you for this comment, we have addressed each comment and suggestion which has allowed us the opportunity to make the protocol clearer overall.

Reviewer 2 comment 2:

*“Scope and heterogeneity of ABI. ABI is an extremely broad and heterogeneous construct encompassing multiple, distinct diagnostic groups. Attempting to summarize incidence and prevalence for all ABI subtypes and severities within a single review may be overly ambitious and risk estimates that are too difficult to interpret. In particular, combining traumatic and non traumatic/atraumatic etiologies may obscure important differences in mechanisms, risk factors, and outcomes. The authors should consider narrowing the scope, for example, separating TBI and ABI, or, at minimum, planning clearly separated analyses and reporting for these categories.”*

Author reply to reviewer 2 comment 2:

Thank you for this important comment. We agree that our original wording could be interpreted as encompassing a very broad range of acquired brain injury (ABI) aetiologies, potentially increasing clinical heterogeneity and limiting comparability across studies. To address this, we have strengthened the eligibility criteria by providing an explicit operational definition of ABI and specifying the diagnostic categories that will be included in the review, please see pages 7-8, specifically lines 147-168.

For the purposes of this review, ABI is defined as a brain injury occurring after birth resulting from traumatic or non-congenital, non-degenerative pathological processes. Eligible ABI categories are limited to traumatic brain injury (TBI), stroke (ischaemic and haemorrhagic), encephalitis, and brain tumour-related brain injury. These categories were selected because they represent major causes of childhood ABI with established epidemiological literature and are sufficiently identifiable within population-based studies. We have also clarified that congenital brain abnormalities, genetic and neurodevelopmental disorders, perinatal and birth-related brain injuries, and progressive neurodegenerative conditions are excluded. This revised definition reduces heterogeneity and improves transparency regarding the scope of the review.

Additionally, in reference to the comment regarding the potential issue of combining traumatic and non-traumatic/traumatic aetiologies, we thank the reviewer for this comment and have addressed this by making the criteria for inclusion in the meta-analysis clearer within the data synthesis section of the methods specifically page 13-14 line 322-377. In summary, we will not pool estimates across all ABI aetiologies, as this would increase the heterogeneity of the estimates; rather we will only conduct a meta-analysis on studies that report statistics or clinically relevant details for the same ABI aetiology, for a meta-analysis to be conducted, a minimum of 2 studies will be required.

Reviewer 2 comment 3:

*“2. Insufficiently defined inclusion criteria for ABI. Similar to above, the current inclusion criteria for ABI does not provide a clear list of diagnoses (e.g., traumatic, hypoxic–ischemic, infectious, vascular, toxic, neoplastic, etc.). As written, the criteria is very broad, which is likely to yield a highly heterogeneous set of conditions and case definitions, limiting comparability across studies and the validity of pooled estimates. The authors should articulate a more explicit operational definition of ABI and specify which diagnostic categories and etiologies are in scope.”*

Author reply to reviewer 2 comment 3:

Thank you for this comment. As noted in our response to the previous comment, we have revised the eligibility criteria to provide a clearer operational definition of acquired brain injury (ABI) and to explicitly define the aetiologies included in the review. To improve consistency and reduce heterogeneity, ABI is now restricted to four predefined categories: traumatic brain injury, stroke (ischaemic and haemorrhagic), encephalitis, and brain tumour-related brain injury. We have also clarified that congenital, genetic, neurodevelopmental, birth-related, and prematurity-related brain injuries are excluded. Although ABI can encompass other aetiologies (e.g., hypoxic-ischaemic, toxic, or metabolic injuries), these are outside the scope of the present review to ensure comparability across studies and validity of pooled estimates. The manuscript has been amended accordingly, please see pages 8 specifically lines 158-168.

Reviewer 2 comment 4:

*“3. Definition and scope of “outcomes”. The term “outcome” is currently under-specified. Given the clinical importance of ABI in childhood, it would be valuable to define outcomes more comprehensively, including cognitive, functional, and behavioral outcomes where available. At the same time, the authors should acknowledge that identifying and synthesizing consistent outcome measures across such a heterogeneous group may be challenging and may require a more focused outcome framework or determine specific outcome domains to target.”*

Author reply to reviewer 2 comment 4:

Thank you for this comment. To clarify the outcomes for this systematic review and meta-analysis will be acquired brain injury, specifically traumatic brain injury, brain tumor, encephalitis, and stroke, within the 0–18-year-old population; measured as incidence and/or prevalence. As noted in our responses to reviewer 2 comments 2 and 3, we have revised the definitions of ABI for this study, which has allowed us to clarify the target outcomes for this review; please see the ‘outcomes’ section on pages 7-8, specifically lines 146-168.

Reviewer 2 comment 5:

“Minor concerns

1. Severity definition and comparability. Severity is not well defined in the current protocol, which likely reflects the broad and heterogeneous nature of the proposed ABI group. Different studies may use different severity metrics (e.g., GCS for TBI, imaging findings, ICU admission, or other proxies), and these are not directly comparable across etiologies. In addition, variables such as length of stay are influenced by many non clinical factors and are not specific indicators of injury or disease severity.”

Author reply to reviewer 2 comment 5:

Thank you for this comment, we agree that severity had not been defined well in this protocol and we thank you for the opportunity to clarify this. In terms of severity metrics in ABI research, metrics are typically inconsistently captured; because of this, in this review we will only pool estimates of studies that have comparable metrics, and where not appropriate we will describe the studies narratively. This has been added to the protocol on page 14 lines 370-377.

Reviewer 2 comment 6:

2. *Exclusion of non–population-based clinical cohorts. The requirement for population-based incidence/prevalence estimates may potentially exclude single center and multi center hospital cohorts. While methodologically understandable, this approach may omit important information on ABI patterns in settings where population registries are limited, particularly in low and middle income countries.*

Author reply to reviewer 2 comment 6:

Thank you for this comment. We agree that there will be an exclusion of non-population-based clinical cohorts and have identified this as a limitation of the review. However, the purpose of our review is to summarise the available evidence that specifically describes a population-based risk and we recognise that this will exclude important single centres where these do not have a clear catchment and that this will also vary by setting. We have explained this on page 8-9 lines 175-196 but have also now added this to the limitations on page 14-15 lined 385-442. We will ensure we describe in the limitations of the final systematic review that exclusion of such studies will be a limitation. This review will also highlight these data gaps that should be recognised to improve and increase the case for further research and robust surveillance in all regions.

Reviewer 2 comment 7:

3. *Justification for the year 2000 cut off. The choice of 2000 as the lower bound for included data is not currently justified. The authors should provide a rationale for this temporal cut off, such as changes in diagnostic practices, coding systems, trauma systems, etc. and discuss how this may affect comparability with older studies.*

Author reply to reviewer 2 comment 7:

Thank you for your comment. We have taken the opportunity to expand upon our justification, explaining that this decision was made to align with current clinical settings and that there was very little digital data capture before the year 2000. These changes can be seen on page 9 lines 198-201.

Reviewer 2 comment 8:

4. Handling of age cut offs and age bands. Although the overall age range (0–18 years) is clearly stated, it is not clear how the authors will handle age bands reported in the primary studies. It will be important to specify how age brackets will be defined, how diagnoses will be grouped within age strata, and what will be done when age categories do not align exactly with the 0–18 range. Clarifying these procedures will improve transparency and interpretability of age specific estimates.

Author reply to reviewer 2 comment 8:

Thank you for this comment. We recognise that we could have been clearer about how age pooling would work. We have taken this opportunity to explain that we will be using developmental milestone ages to separate the age bands for pooling; this was decided due to the changing injury mechanism prevalence seen in the literature as a child ages. If a study does not report their data in the same way as our predefined age brackets, they will be narratively described rather than included in the sub-group analysis. The changes made in response to this can be seen on page 14, lines 363-369.

Reviewer 3 comment 1:

*This protocol addresses an important and underexplored topic: the global epidemiology of acquired brain injury (ABI) in children and young people. The authors aim to synthesize incidence and prevalence estimates across traumatic and non-traumatic etiologies using systematic review and meta-analysis. The topic is clinically relevant and may inform rehabilitation services, health policy, and future epidemiologic research.*

Author reply to reviewer 3 comment 1:

Thank you for this comment, we agree that this review will explore an important and underexplained topic.

Reviewer 3 comment 2:

*The manuscript is generally well designed, with plans to follow PRISMA-P conventions and several methodological strengths. However, important conceptual and methodological limitations arise from a lack of clarity on the definition of “acquired brain injury” and the disorders the authors plan to include in the analysis.*

*In its current form, using “acquired brain injury” as a common measurable outcome across highly diverse diseases, populations, healthcare systems, and resource settings risks producing pooled estimates that may be difficult to interpret and synthesize.*

Author reply to reviewer 3 comment 2:

Thank you for this comment; we recognise that in the submitted manuscript there was a lack of clarity surrounding the definition of ABI. We have, as a result, made our definitions clearer in our outcomes section (specifically page 7-8 lines 146-168). Furthermore, in response to similar comments surrounding the difficulty of pooling estimates, we have made our explanations clearer within the data synthesis section on paged 13-14, lines 322-377.

Reviewer 3 comment 3:

*Some comments the authors may wish to consider in refining the final protocol:*

*How will the review distinguish acute neurologic injury from chronic neurologic sequelae, and how will secondary injury processes be handled?*

Author reply to reviewer 3 comment 3:

Thank you for this comment. The primary outcome of this review is the occurrence of acquired brain injury (ABI), defined by the presence of a specified ABI diagnosis rather than its subsequent long-term consequences. We have clarified that studies will be eligible if they report the incidence or prevalence of ABI diagnoses (traumatic brain injury, stroke, encephalitis, or brain tumour-related brain injury) within a defined population. Studies reporting only chronic neurological sequelae, functional impairments, or rehabilitation outcomes without ascertainment of the underlying ABI diagnosis will not be included.

We have also clarified that secondary injury processes (e.g., cerebral oedema, inflammation, seizures, or other complications arising following an ABI) will not be considered separate ABI events. Where reported, these data will be extracted as clinical characteristics or measures of injury severity, rather than as distinct outcomes. This approach ensures that the review focuses on the epidemiology of primary ABI diagnoses while avoiding double-counting of injury-related complications. The changes made in response to this comment can be seen on pages 7-8, lines 147-168.

Reviewer 3 comment 4:

*As this is intended to be a global review, how will country- and region-specific differences in diagnostic methodology, surveillance systems, and healthcare access be accounted for?*

Author reply to reviewer 3 comment 4:

Thank you for this comment. We recognise that differences in diagnostic practices, surveillance systems, and healthcare access may contribute to heterogeneity across countries and regions. While these factors cannot be fully controlled within a systematic review, we will extract detailed information on ABI ascertainment methods and data sources, including population registries, surveillance systems, administrative databases, and clinically confirmed diagnoses. The items for extraction can be seen on page 11-12 lines 279-314. Where sufficient data are available, we will explore these factors through subgroup analyses and meta-regression. We will also report findings by country and geographic region and only pool studies where case definitions and data collection methods are sufficiently comparable. Where meaningful quantitative synthesis is not appropriate, findings will be presented narratively. This approach will help distinguish methodological variation from true differences in ABI occurrence. This is reflected in the manuscript on page 13-14 lines 339-377.

Reviewer 3 comment 5:

*How do the authors plan to harmonize the substantial heterogeneity between infectious neurologic disorders, traumatic brain injuries, neoplastic conditions, and chronic sequelae of acute neurologic insults?*

*These differences in disease manifestation and duration will also affect the interpretation of incidence and prevalence estimates and the question of whether conditions can reasonably be categorized together.*

Author reply to reviewer 3 comment 5:

Thank you for these comments, we agree that ABI encompasses conditions with differing pathophysiology, clinical presentation, and duration, which may influence both incidence and prevalence estimates. To address this, we will not assume that all ABI categories are directly comparable. As described in response to the previous comments, analyses will be conducted separately by ABI subtype, and pooled estimates will only be generated where studies are sufficiently comparable in terms of diagnosis, case definition, and methodology. We will assess heterogeneity using subgroup analyses and meta-regression where data permit, and where meaningful quantitative synthesis is not appropriate, findings will be presented narratively. This approach will allow differences between ABI subtypes and disease trajectories to be considered during interpretation of the findings.

Reviewer 3 comment 6:

*The background synthesis appears more strongly focused on traumatic brain injury (TBI) epidemiology than on ABI broadly defined. If TBI is the principal intended focus, narrowing the scope accordingly may improve the interpretability and feasibility of quantitative synthesis and pooled meta-analysis.*

Author reply to reviewer 3 comment 6:

Thank you for this comment, whilst we do agree that there is a heavy focus on TBI in the background synthesis in comparison to other ABI injury types, we have provided background on the other injury types focused on for this review. We have taken this opportunity to add in more explanation surrounding the literature discrepancy focused on alternative ABI types as opposed to TBI. This can be seen on page 5, lines 96-100.

Reviewer 3 comment 7:

*Minor comment:*

*The authors may wish to further explain the use of the term “silent epidemic” in relation to the aims and epidemiologic scope of this review.*

Author reply to reviewer 3 comment 7:

Thank you for this comment. We have taken the opportunity to add in a brief explanation of how the term silent epidemic relates to this review, namely the lack of incidence and prevalence estimations for children and young people which also related to underdiagnosis and late diagnosis. We have provided references to support this explanation. Please see page 4 lines 72-74.

Closing summary:

We thank all reviewers again for their constructive feedback, which has helped us to improve our manuscript. We believe the revised manuscript addresses all concerns raised and hope it is now suitable for publication.

Sincerely,

**Laila J Tata**

**Professor of Epidemiology**

**University of Nottingham**

School of Medicine

Lifespan and Population Health Unit

Clinical Sciences Building (Room B43)

City Hospital Campus

Hucknall Road

University of Nottingham

Nottingham NG5 1PB

United Kingdom
