# Supplementary material for: Incidence and prevalence of acquired brain injury in children and young people globally: A systematic review and meta-analysis protocol
Source: PLoS One. 2026 Oct 8;21(10):e0357762. doi: 10.1371/journal.pone.0357762 (PMC13648995; doi:10.1371/journal.pone.0357762)
Supplement: Peer review history

# Peer review: Decision Letter 0

**DOI:** 10.1371/journal.pone.0357762.r001

**Authors:** Firas H Kobeissy

3 Jun 2026

Dear Dr. Orchard,

Thank you for submitting your manuscript to PLOS ONE. After careful consideration, we feel that it has merit but does not fully meet PLOS ONE’s publication criteria as it currently stands. Therefore, we invite you to submit a revised version of the manuscript that addresses the points raised during the review process.

Please submit your revised manuscript by Jul 18 2026 11:59PM. If you will need more time than this to complete your revisions, please reply to this message or contact the journal office at plosone@plos.org. When you're ready to submit your revision, log on to https://www.editorialmanager.com/pone/ and select the 'Submissions Needing Revision' folder to locate your manuscript file.

- A letter that responds to each point raised by the academic editor and reviewer(s). You should upload this letter as a separate file labeled 'Response to Reviewers'.
- A marked-up copy of your manuscript that highlights changes made to the original version. You should upload this as a separate file labeled 'Revised Manuscript with Track Changes'.
- An unmarked version of your revised paper without tracked changes. You should upload this as a separate file labeled 'Manuscript'.

If you would like to make changes to your financial disclosure, please include your updated statement in your cover letter. Guidelines for resubmitting your figure files are available below the reviewer comments at the end of this letter.

If applicable, we recommend that you deposit your laboratory protocols in protocols.io to enhance the reproducibility of your results. Protocols.io assigns your protocol its own identifier (DOI) so that it can be cited independently in the future. For instructions see: https://journals.plos.org/plosone/s/submission-guidelines#loc-laboratory-protocols. Additionally, PLOS ONE offers an option for publishing peer-reviewed Lab Protocol articles, which describe protocols hosted on protocols.io. Read more information on sharing protocols at https://plos.org/protocols?utm_medium=editorial-email&utm_source=authorletters&utm_campaign=protocols.

As the corresponding author, your ORCID iD is verified in the submission system and will appear in the published article. PLOS supports the use of ORCID, and we encourage all coauthors to register for an ORCID iD and use it as well. Please encourage your coauthors to verify their ORCID iD within the submission system before final acceptance, as unverified ORCID iDs will not appear in the published article. *Only*  the individual author can complete the verification step; PLOS staff *cannot*  verify ORCID iDs on behalf of authors.

We look forward to receiving your revised manuscript.

Kind regards,

Firas H Kobeissy, PhD

Academic Editor

PLOS One

Journal Requirements:

When submitting your revision, we need you to address these additional requirements.

1. Please ensure that your manuscript meets PLOS ONE's style requirements, including those for file naming. The PLOS ONE style templates can be found at https://journals.plos.org/plosone/s/file?id=wjVg/PLOSOne_formatting_sample_main_body.pdf and https://journals.plos.org/plosone/s/file?id=ba62/PLOSOne_formatting_sample_title_authors_affiliations.pdf

2. Thank you for stating the following financial disclosure:

[This research was funded by the Eveline Walker Brain Injury Research fund through a grant to the University of Nottingham (PR11128), the NIHR Applied Research Collaboration East Midlands (ARC EM, NIHR200171), the NIHR Biomedical Research Centre (Nottingham, NIHR203310), and the National Rehabilitation Centre. The views expressed are those of the author(s) and not necessarily those of the NIHR, the Department of Health and Social Care or any of the other co-funders.].

Please state what role the funders took in the study.  If the funders had no role, please state: "The funders had no role in study design, data collection and analysis, decision to publish, or preparation of the manuscript."

If this statement is not correct you must amend it as needed.

Please include this amended Role of Funder statement in your cover letter; we will change the online submission form on your behalf.

If the reviewer comments include a recommendation to cite specific previously published works, please review and evaluate these publications to determine whether they are relevant and should be cited. There is no requirement to cite these works unless the editor has indicated otherwise.

[Note: HTML markup is below. Please do not edit.]

Reviewers' comments:

Reviewer's Responses to Questions

**Comments to the Author**

1. Does the manuscript provide a valid rationale for the proposed study, with clearly identified and justified research questions?

Reviewer #1: Yes

Reviewer #2: Partly

Reviewer #3: Yes

Reviewer #4: Yes

**********

2. Is the protocol technically sound and planned in a manner that will lead to a meaningful outcome and allow testing the stated hypotheses?

Reviewer #1: Yes

Reviewer #2: Partly

Reviewer #3: Partly

Reviewer #4: Partly

**********

3. Is the methodology feasible and described in sufficient detail to allow the work to be replicable?

Reviewer #1: Yes

Reviewer #2: Yes

Reviewer #3: Yes

Reviewer #4: Yes

**********

4. Have the authors described where all data underlying the findings will be made available when the study is complete?

The PLOS Data policy

Reviewer #1: Yes

Reviewer #2: Yes

Reviewer #3: Yes

Reviewer #4: No

**********

5. Is the manuscript presented in an intelligible fashion and written in standard English?

Reviewer #1: Yes

Reviewer #2: Yes

Reviewer #3: Yes

Reviewer #4: Yes

**********

Please use the space provided to explain your answers to the questions above and, if applicable, provide comments about issues authors must address before this protocol can be accepted for publication. You may also include additional comments for the author, including concerns about research or publication ethics.

You may also provide optional suggestions and comments to authors that they might find helpful in planning their study.

Reviewer #1: This is a well-structured and timely protocol addressing an important clinical and public health gap. The manuscript clearly articulates the rationale for a global synthesis of childhood acquired brain injury epidemiology, and the methodological approach is rigorous and appropriate for a systematic review and meta-analysis.

The protocol is comprehensive in scope, incorporating both traumatic and non-traumatic brain injuries across multiple subtypes (TBI, stroke, tumors, encephalitis). The methods are detailed and transparent, with appropriate use of established frameworks (PRISMA-P and PRISMA-S guidelines, JBI Critical Appraisal Checklist). The planned subgroup analyses by age, geography, ethnicity, and socioeconomic factors will provide valuable granularity for identifying disparities and service gaps. Registration with PROSPERO and the inclusion of grey literature strengthen reproducibility and reduce bias. The clinical significance is clear, as quantifying the global burden will meaningfully inform service planning and policy.

In the Study Records/Selection Process section, it is stated that conflicts between reviewers will be resolved through discussion or consultation with a third team member. Consider briefly specifying a priori decision rules or criteria that will guide these discussions (for example, how borderline cases at the inclusion/exclusion threshold will be handled, or whether specific study characteristics will trigger mandatory third-reviewer consultation). This adds an additional layer of transparency and helps prevent post-hoc decision-making during screening.

Reviewer #2: This systematic review and meta-analysis addresses an important and understudied topic. The focus on population-based data and contemporary estimates has the potential to provide valuable information for healthcare and service planning as well as policy for children with ABI. The manuscript is clearly written, the age range is well specified, and there is a thoughtful attempt to consider ABI across subtypes and severities. With some refinement of the scope and clearer operational definitions, this protocol and eventual study could make a substantial contribution to pediatric ABI literature. Below are specific comments and suggestions.

Primary concerns

1. Scope and heterogeneity of ABI. ABI is an extremely broad and heterogeneous construct encompassing multiple, distinct diagnostic groups. Attempting to summarize incidence and prevalence for all ABI subtypes and severities within a single review may be overly ambitious and risk estimates that are too difficult to interpret. In particular, combining traumatic and non traumatic/atraumatic etiologies may obscure important differences in mechanisms, risk factors, and outcomes. The authors should consider narrowing the scope, for example, separating TBI and ABI, or, at minimum, planning clearly separated analyses and reporting for these categories.

2. Insufficiently defined inclusion criteria for ABI. Similar to above, the current inclusion criteria for ABI does not provide a clear list of diagnoses (e.g., traumatic, hypoxic–ischemic, infectious, vascular, toxic, neoplastic, etc.). As written, the criteria is very broad, which is likely to yield a highly heterogeneous set of conditions and case definitions, limiting comparability across studies and the validity of pooled estimates. The authors should articulate a more explicit operational definition of ABI and specify which diagnostic categories and etiologies are in scope.

3. Definition and scope of “outcomes”. The term “outcome” is currently under-specified. Given the clinical importance of ABI in childhood, it would be valuable to define outcomes more comprehensively, including cognitive, functional, and behavioral outcomes where available. At the same time, the authors should acknowledge that identifying and synthesizing consistent outcome measures across such a heterogeneous group may be challenging and may require a more focused outcome framework or determine specific outcome domains to target.

Minor concerns

1. Severity definition and comparability. Severity is not well defined in the current protocol, which likely reflects the broad and heterogeneous nature of the proposed ABI group. Different studies may use different severity metrics (e.g., GCS for TBI, imaging findings, ICU admission, or other proxies), and these are not directly comparable across etiologies. In addition, variables such as length of stay are influenced by many non clinical factors and are not specific indicators of injury or disease severity.

2. Exclusion of non–population-based clinical cohorts. The requirement for population-based incidence/prevalence estimates may potentially exclude single center and multi center hospital cohorts. While methodologically understandable, this approach may omit important information on ABI patterns in settings where population registries are limited, particularly in low and middle income countries.

3. Justification for the year 2000 cut off. The choice of 2000 as the lower bound for included data is not currently justified. The authors should provide a rationale for this temporal cut off, such as changes in diagnostic practices, coding systems, trauma systems, etc. and discuss how this may affect comparability with older studies.

4. Handling of age cut offs and age bands. Although the overall age range (0–18 years) is clearly stated, it is not clear how the authors will handle age bands reported in the primary studies. It will be important to specify how age brackets will be defined, how diagnoses will be grouped within age strata, and what will be done when age categories do not align exactly with the 0–18 range. Clarifying these procedures will improve transparency and interpretability of age specific estimates.

Reviewer #3: This protocol addresses an important and underexplored topic: the global epidemiology of acquired brain injury (ABI) in children and young people. The authors aim to synthesize incidence and prevalence estimates across traumatic and non-traumatic etiologies using systematic review and meta-analysis. The topic is clinically relevant and may inform rehabilitation services, health policy, and future epidemiologic research.

The manuscript is generally well designed, with plans to follow PRISMA-P conventions and several methodological strengths. However, important conceptual and methodological limitations arise from a lack of clarity on the definition of “acquired brain injury” and the disorders the authors plan to include in the analysis.

In its current form, using “acquired brain injury” as a common measurable outcome across highly diverse diseases, populations, healthcare systems, and resource settings risks producing pooled estimates that may be difficult to interpret and synthesize.

Some comments the authors may wish to consider in refining the final protocol:

1. How will the review distinguish acute neurologic injury from chronic neurologic sequelae, and how will secondary injury processes be handled?

2. As this is intended to be a global review, how will country- and region-specific differences in diagnostic methodology, surveillance systems, and healthcare access be accounted for?

3. How do the authors plan to harmonize the substantial heterogeneity between infectious neurologic disorders, traumatic brain injuries, neoplastic conditions, and chronic sequelae of acute neurologic insults?

4. These differences in disease manifestation and duration will also affect the interpretation of incidence and prevalence estimates and the question of whether conditions can reasonably be categorized together.

The background synthesis appears more strongly focused on traumatic brain injury (TBI) epidemiology than on ABI broadly defined. If TBI is the principal intended focus, narrowing the scope accordingly may improve the interpretability and feasibility of quantitative synthesis and pooled meta-analysis.

Minor comment:

The authors may wish to further explain the use of the term “silent epidemic” in relation to the aims and epidemiologic scope of this review.

Reviewer #4: 1. The manuscript addresses a highly relevant, clinically significant issue: the lack of effective targeted treatments for long-term cognitive impairment following traumatic brain injury (TBI). The authors build a cohesive logical foundation by combining established observations: that post-TBI glymphatic dysfunction drives toxic protein clearance failures (p-tau and Aβ accumulation) , and that β-blockers like propranolol exhibit neuroprotective and mortality benefits in clinical datasets. By exploring whether propranolol clears toxic aggregates and rescues spatial memory specifically via the astrocytic STAT3/MMP2/AQP4 molecular axis, the study addresses an important mechanistic gap in neurotrauma pharmacology.

2. The primary experimental pipeline is robustly conceived, applying a controlled cortical impact (CCI) model in vivo, followed by a solid array of behavioral testing (Novel Object Recognition, Odor Test, Morris Water Maze) , ex vivo glymphatic tracing, and in vitro mechanistic verification in primary astrocytes. Crucially, the authors addressed a major initial flaw by incorporating a healthy Normal Control group into their revised data , allowing them to recognize that while propranolol substantially improves memory performance and clears p-tau/Aβ , it does not completely return the injured brain to normal baseline parameters. However, unresolved issues remain that weaken the overall technical soundness. First, the authors explicitly state in their limitations that high-dose propranolol appeared to harm cognitive function, yet this critical data is completely omitted from the text. To make the outcome truly meaningful and robust, this dose-response curve must be explicitly illustrated and statistically compared.

3. The methods described are reproducible. The authors supplied specific details for the intracisternal glymphatic tracking protocol, specifying stereotaxic fixation, the use of a 30GA needle, the molecular weight of the fluorescent tracer, specific pump infusion metrics, precise tissue harvesting timepoints (30 minutes post-infusion), and the imaging/quantification systems utilized (Zeiss LSM 510 NLO and MCID software). The cell culture conditions—challenging astrocytes with noradrenaline and rescuing with propranolol—are structurally clear and replicable.

4. The manuscript text does not explicitly feature a designated Data Availability Statement detailing where the raw individual data points, western blot source gels, or full-sized high-resolution confocal micrographs will be publically deposited. Furthermore, the inclusion of statements such as high-dose toxicity data being left out as "(data not seen)" runs counter to the PLOS Data Policy, which mandates that all data underlying findings or claims must be accessible. The authors must declare a public repository (e.g., Dryad, Figshare, or Zenodo) for their complete underlying dataset

5. While the overall narrative is comprehensible, the manuscript contains persistent structural, grammatical, and typographical errors that fall short of professional publication standards and lack clarity. Hence, the manuscript could benefit from proofreading and structural polishing. Some examples of specific errors include:

* Awkward Phrasing and Syntactical Errors: Sentences frequently use non-standard phrasing such as: "These are numbers of proteinaceous waste products..." , "The level of p-STAT3 and MMP2 were knockdown...", "The vitro research showed..." (should be In vitro) , and "...how the correction will meet with approval.".

* Capitalization and Nomenclature Fluctuations: Inconsistent capitalization occurs inside text bodies, such as capitalizing the United States as "United STATes" in the bibliography. The text also intermixes the terms "glymphatic system" and "lymphoid system" interchangeably when discussing central nervous system fluid clearance, which is conceptually confusing.

**********

what does this mean?). If published, this will include your full peer review and any attached files.

If you choose “no”, your identity will remain anonymous but your review may still be made public.

**Do you want your identity to be public for this peer review?**  For information about this choice, including consent withdrawal, please see our Privacy Policy

Reviewer #1: **Yes:** Jessica Wright

Reviewer #2: No

Reviewer #3: No

Reviewer #4: No

**********

[NOTE: If reviewer comments were submitted as an attachment file, they will be attached to this email and accessible via the submission site. Please log into your account, locate the manuscript record, and check for the action link "View Attachments". If this link does not appear, there are no attachment files.]

To ensure your figures meet our technical requirements, please review our figure guidelines: https://journals.plos.org/plosone/s/figures

You may also use PLOS’s free figure tool, NAAS, to help you prepare publication quality figures: https://journals.plos.org/plosone/s/figures#loc-tools-for-figure-preparation.

NAAS will assess whether your figures meet our technical requirements by comparing each figure against our figure specifications.

# Author comment: Author response to Decision Letter 1

**DOI:** 10.1371/journal.pone.0357762.r002

4 Aug 2026

Dear PLoS One editor and reviewers,

We would like to thank both the editor and the reviewers for taking the time to read and review our manuscript, and for providing constructive feedback. We have revised the manuscript in response to your comments and have provided a point-by-point response below.

For ease, we have italicised the reviewers’ comments, and our author responses follow in plain text; we have also added page number and line numbers within responses where appropriate that relate to the revised manuscript.

Reviewer 1 comment 1:

“This is a well-structured and timely protocol addressing an important clinical and public health gap. The manuscript clearly articulates the rationale for a global synthesis of childhood acquired brain injury epidemiology, and the methodological approach is rigorous and appropriate for a systematic review and meta-analysis.

The protocol is comprehensive in scope, incorporating both traumatic and non-traumatic brain injuries across multiple subtypes (TBI, stroke, tumors, encephalitis). The methods are detailed and transparent, with appropriate use of established frameworks (PRISMA-P and PRISMA-S guidelines, JBI Critical Appraisal Checklist). The planned subgroup analyses by age, geography, ethnicity, and socioeconomic factors will provide valuable granularity for identifying disparities and service gaps. Registration with PROSPERO and the inclusion of grey literature strengthen reproducibility and reduce bias. The clinical significance is clear, as quantifying the global burden will meaningfully inform service planning and policy.”

Author reply to reviewer 1 comment 1:

We thank the reviewer for the positive comments surrounding the relevance of our protocol. We agree and thank them also for their recollection of the robust methodology presented and have retained this in the revised manuscript.

Reviewer 1 comment 2:

“In the Study Records/Selection Process section, it is stated that conflicts between reviewers will be resolved through discussion or consultation with a third team member. Consider briefly specifying a priori decision rules or criteria that will guide these discussions (for example, how borderline cases at the inclusion/exclusion threshold will be handled, or whether specific study characteristics will trigger mandatory third-reviewer consultation). This adds an additional layer of transparency and helps prevent post-hoc decision-making during screening.”

Author reply to reviewer 1 comment 2:

Thank you for this suggestion. We have now added on page 10-11 lines 226-270 a description of how a third reviewer may be used, this is as follows: “Following de-duplication, dual screening will be used for study inclusion in two stages. For stage 1, title and abstract screening, SO will screen all studies, and a team of secondary reviewers will each screen 20% of all studies. To proceed to the full text screening stage, a study should meet the following criteria: 1) report an incidence and/or prevalence of ABI overall or at least one of the specified ABI diagnoses (traumatic brain injury, stroke, encephalitis, or brain tumour) or data to calculate these, 2) the study aimed to obtain a representative sample of children with ABI whose injuries occurred between 0-18 years old, and 3) the children in the baseline represent a general population-based study sample or externally available relevant general-population data are used with a clearly referenced source. Any uncertainty or disagreement between reviewers arises will be discussed to resolve screening decisions, however, if agreement cannot be met on these criteria, a third reviewer will independently screen the study and make a decision; the study will proceed to full text for review of key details if any uncertainty remains. For stage 2, all studies reaching the full text screening stage will all be dual screened by the reviewing team. To be included in the final review and proceed to data extraction the following will be checked to ensure they fully meet the inclusion criteria: 1) if any incidence/prevalence data is collected pre-2000, the 2000-onwards data is separately extractable, 2) if any incidence/prevalence data is collected on a population without age restriction, that any data for children between age 0-18 is separately extractable, and 3) each study must report the number of children and young people with ABI overall or at least one of the ABI diagnoses, and report the number of children in the denominator used for incidence and/or prevalence calculations (if one or both of these details are missing, the author will be contacted for these details). If any conflicts arise at this stage, reviewers will meet to discuss the conflicts and review the paper together; if agreement cannot be met based on the inclusion criteria, a third reviewer will be sought to make the decision..”

Reviewer 2 comment 1:

“This systematic review and meta-analysis addresses an important and understudied topic. The focus on population-based data and contemporary estimates has the potential to provide valuable information for healthcare and service planning as well as policy for children with ABI. The manuscript is clearly written, the age range is well specified, and there is a thoughtful attempt to consider ABI across subtypes and severities. With some refinement of the scope and clearer operational definitions, this protocol and eventual study could make a substantial contribution to pediatric ABI literature. Below are specific comments and suggestions.”

Author reply to reviewer 2 comment 1:

Thank you for this comment, we have addressed each comment and suggestion which has allowed us the opportunity to make the protocol clearer overall.

Reviewer 2 comment 2:

“Scope and heterogeneity of ABI. ABI is an extremely broad and heterogeneous construct encompassing multiple, distinct diagnostic groups. Attempting to summarize incidence and prevalence for all ABI subtypes and severities within a single review may be overly ambitious and risk estimates that are too difficult to interpret. In particular, combining traumatic and non traumatic/atraumatic etiologies may obscure important differences in mechanisms, risk factors, and outcomes. The authors should consider narrowing the scope, for example, separating TBI and ABI, or, at minimum, planning clearly separated analyses and reporting for these categories.”

Author reply to reviewer 2 comment 2:

Thank you for this important comment. We agree that our original wording could be interpreted as encompassing a very broad range of acquired brain injury (ABI) aetiologies, potentially increasing clinical heterogeneity and limiting comparability across studies. To address this, we have strengthened the eligibility criteria by providing an explicit operational definition of ABI and specifying the diagnostic categories that will be included in the review, please see pages 7-8, specifically lines 147-168.

For the purposes of this review, ABI is defined as a brain injury occurring after birth resulting from traumatic or non-congenital, non-degenerative pathological processes. Eligible ABI categories are limited to traumatic brain injury (TBI), stroke (ischaemic and haemorrhagic), encephalitis, and brain tumour-related brain injury. These categories were selected because they represent major causes of childhood ABI with established epidemiological literature and are sufficiently identifiable within population-based studies. We have also clarified that congenital brain abnormalities, genetic and neurodevelopmental disorders, perinatal and birth-related brain injuries, and progressive neurodegenerative conditions are excluded. This revised definition reduces heterogeneity and improves transparency regarding the scope of the review.

Additionally, in reference to the comment regarding the potential issue of combining traumatic and non-traumatic/traumatic aetiologies, we thank the reviewer for this comment and have addressed this by making the criteria for inclusion in the meta-analysis clearer within the data synthesis section of the methods specifically page 13-14 line 322-377. In summary, we will not pool estimates across all ABI aetiologies, as this would increase the heterogeneity of the estimates; rather we will only conduct a meta-analysis on studies that report statistics or clinically relevant details for the same ABI aetiology, for a meta-analysis to be conducted, a minimum of 2 studies will be required.

Reviewer 2 comment 3:

“2. Insufficiently defined inclusion criteria for ABI. Similar to above, the current inclusion criteria for ABI does not provide a clear list of diagnoses (e.g., traumatic, hypoxic–ischemic, infectious, vascular, toxic, neoplastic, etc.). As written, the criteria is very broad, which is likely to yield a highly heterogeneous set of conditions and case definitions, limiting comparability across studies and the validity of pooled estimates. The authors should articulate a more explicit operational definition of ABI and specify which diagnostic categories and etiologies are in scope.”

Author reply to reviewer 2 comment 3:

Thank you for this comment. As noted in our response to the previous comment, we have revised the eligibility criteria to provide a clearer operational definition of acquired brain injury (ABI) and to explicitly define the aetiologies included in the review. To improve consistency and reduce heterogeneity, ABI is now restricted to four predefined categories: traumatic brain injury, stroke (ischaemic and haemorrhagic), encephalitis, and brain tumour-related brain injury. We have also clarified that congenital, genetic, neurodevelopmental, birth-related, and prematurity-related brain injuries are excluded. Although ABI can encompass other aetiologies (e.g., hypoxic-ischaemic, toxic, or metabolic injuries), these are outside the scope of the present review to ensure comparability across studies and validity of pooled estimates. The manuscript has been amended accordingly, please see pages 8 specifically lines 158-168.

Reviewer 2 comment 4:

“3. Definition and scope of “outcomes”. The term “outcome” is currently under-specified. Given the clinical importance of ABI in childhood, it would be valuable to define outcomes more comprehensively, including cognitive, functional, and behavioral outcomes where available. At the same time, the authors should acknowledge that identifying and synthesizing consistent outcome measures across such a heterogeneous group may be challenging and may require a more focused outcome framework or determine specific outcome domains to target.”

Author reply to reviewer 2 comment 4:

Thank you for this comment. To clarify the outcomes for this systematic review and meta-analysis will be acquired brain injury, specifically traumatic brain injury, brain tumor, encephalitis, and stroke, within the 0–18-year-old population; measured as incidence and/or prevalence. As noted in our responses to reviewer 2 comments 2 and 3, we have revised the definitions of ABI for this study, which has allowed us to clarify the target outcomes for this review; please see the ‘outcomes’ section on pages 7-8, specifically lines 146-168.

Reviewer 2 comment 5:

“Minor concerns

1. Severity definition and comparability. Severity is not well defined in the current protocol, which likely reflects the broad and heterogeneous nature of the proposed ABI group. Different studies may use different severity metrics (e.g., GCS for TBI, imaging findings, ICU admission, or other proxies), and these are not directly comparable across etiologies. In addition, variables such as length of stay are influenced by many non clinical factors and are not specific indicators of injury or disease severity.”

Author reply to reviewer 2 comment 5:

Thank you for this comment, we agree that severity had not been defined well in this protocol and we thank you for the opportunity to clarify this. In terms of severity metrics in ABI research, metrics are typically inconsistently captured; because of this, in this review we will only pool estimates of studies that have comparable metrics, and where not appropriate we will describe the studies narratively. This has been added to the protocol on page 14 lines 370-377.

Reviewer 2 comment 6:

2. Exclusion of non–population-based clinical cohorts. The requirement for population-based incidence/prevalence estimates may potentially exclude single center and multi center hospital cohorts. While methodologically understandable, this approach may omit important information on ABI patterns in settings where population registries are limited, particularly in low and middle income countries.

Author reply to reviewer 2 comment 6:

Thank you for this comment. We agree that there will be an exclusion of non-population-based clinical cohorts and have identified this as a limitation of the review. However, the purpose of our review is to summarise the available evidence that specifically describes a population-based risk and we recognise that this will exclude important single centres where these do not have a clear catchment and that this will also vary by setting. We have explained this on page 8-9 lines 175-196 but have also now added this to the limitations on page 14-15 lined 385-442. We will ensure we describe in the limitations of the final systematic review that exclusion of such studies will be a limitation. This review will also highlight these data gaps that should be recognised to improve and increase the case for further research and robust surveillance in all regions.

Reviewer 2 comment 7:

3. Justification for the year 2000 cut off. The choice of 2000 as the lower bound for included data is not currently justified. The authors should provide a rationale for this temporal cut off, such as changes in diagnostic practices, coding systems, trauma systems, etc. and discuss how this may affect comparability with older studies.

Author reply to reviewer 2 comment 7:

Thank you for your comment. We have taken the opportunity to expand upon our justification, explaining that this decision was made to align with current clinical settings and that there was very little digital data capture before the year 2000. These changes can be seen on page 9 lines 198-201.

Reviewer 2 comment 8:

4. Handling of age cut offs and age bands. Although the overall age range (0–18 years) is clearly stated, it is not clear how the authors will handle age bands reported in the primary studies. It will be important to specify how age brackets will be defined, how diagnoses will be grouped within age strata, and what will be done when age categories do not align exactly with the 0–18 range. Clarifying these procedures will improve transparency and interpretability of age specific estimates.

Author reply to reviewer 2 comment 8:

Thank you for this comment. We recognise that we could have been clearer about how age pooling would work. We have taken this opportunity to explain that we will be using developmental milestone ages to separate the age bands for pooling; this was decided due to the changing injury mechanism prevalence seen in the literature as a child ages. If a study does not report their data in the same way as our predefined age brackets, they will be narratively described rather than included in the sub-group analysis. The changes made in response to this can be seen on page 14, lines 363-369.

Reviewer 3 comment 1:

This protocol addresses an important and underexplored topic: the global epidemiology of acquired brain injury (ABI) in children and young people. The authors aim to synthesize incidence and prevalence estimates across traumatic and non-traumatic etiologies using systematic review and meta-analysis. The topic is clinically relevant and may inform rehabilitation services, health policy, and future epidemiologic research.

Author reply to reviewer 3 comment 1:

Thank you for this comment, we agree that this review will explore an important and underexplained topic.

Reviewer 3 comment 2:

The manuscript is generally well designed, with plans to follow PRISMA-P conventions and several methodological strengths. However, important conceptual and methodological l

Attachment Submitted filename: Response to Reviewers.docx

# Peer review: Decision Letter 1

**DOI:** 10.1371/journal.pone.0357762.r003

**Authors:** Firas H Kobeissy

23 Aug 2026

Incidence and prevalence of acquired brain injury in children and young people globally: a systematic review and meta-analysis protocol

PONE-D-26-19774R1

Dear Dr. Tata,

We’re pleased to inform you that your manuscript has been judged scientifically suitable for publication and will be formally accepted for publication once it meets all outstanding technical requirements.

Within one week, you’ll receive an e-mail detailing the required amendments. When these have been addressed, you’ll receive a formal acceptance letter and your manuscript will be scheduled for publication.

An invoice will be generated when your article is formally accepted. Please note, if your institution has a publishing partnership with PLOS and your article meets the relevant criteria, all or part of your publication costs will be covered. Please make sure your user information is up-to-date by logging into Editorial Manager at Editorial Manager® and clicking the ‘Update My Information' link at the top of the page. For questions related to billing, please contact billing support.

If your institution or institutions have a press office, please notify them about your upcoming paper to help maximize its impact. If they’ll be preparing press materials, please inform our press team as soon as possible -- no later than 48 hours after receiving the formal acceptance. Your manuscript will remain under strict press embargo until 2 pm Eastern Time on the date of publication. For more information, please contact onepress@plos.org.

Kind regards,

Firas H Kobeissy, PhD

Academic Editor

PLOS One

Additional Editor Comments (optional):

Reviewers' comments:

Reviewer's Responses to Questions

**Comments to the Author**

1. Does the manuscript provide a valid rationale for the proposed study, with clearly identified and justified research questions?

Reviewer #3: Yes

**********

2. Is the protocol technically sound and planned in a manner that will lead to a meaningful outcome and allow testing the stated hypotheses?

Reviewer #3: Yes

**********

3. Is the methodology feasible and described in sufficient detail to allow the work to be replicable?

Reviewer #3: Yes

**********

4. Have the authors described where all data underlying the findings will be made available when the study is complete?

The PLOS Data policy

Reviewer #3: Yes

**********

5. Is the manuscript presented in an intelligible fashion and written in standard English?

Reviewer #3: Yes

**********

Please use the space provided to explain your answers to the questions above and, if applicable, provide comments about issues authors must address before this protocol can be accepted for publication. You may also include additional comments for the author, including concerns about research or publication ethics.

You may also provide optional suggestions and comments to authors that they might find helpful in planning their study.

Reviewer #3: The revisions and reviewer responses are satisfactory. There are page and line number errors in the response that should be checked against the final manuscript PDF

**********

what does this mean?). If published, this will include your full peer review and any attached files.

If you choose “no”, your identity will remain anonymous but your review may still be made public.

**Do you want your identity to be public for this peer review?**  For information about this choice, including consent withdrawal, please see our Privacy Policy

Reviewer #3: No

**********

# Editor report: Acceptance letter

**DOI:** 10.1371/journal.pone.0357762.r004

**Authors:** Firas H Kobeissy

PONE-D-26-19774R1

PLOS One

Dear Dr. Tata,

I'm pleased to inform you that your manuscript has been deemed suitable for publication in PLOS One. Congratulations! Your manuscript is now being handed over to our production team.

At this stage, our production department will prepare your paper for publication. This includes ensuring the following:

* All references, tables, and figures are properly cited

* All relevant supporting information is included in the manuscript submission,

* There are no issues that prevent the paper from being properly typeset

You will receive further instructions from the production team, including instructions on how to review your proof when it is ready. Please keep in mind that we are working through a large volume of accepted articles, so please give us a few days to review your paper and let you know the next and final steps.

Lastly, if your institution or institutions have a press office, please let them know about your upcoming paper now to help maximize its impact. If they'll be preparing press materials, please inform our press team within the next 48 hours. Your manuscript will remain under strict press embargo until 2 pm Eastern Time on the date of publication. For more information, please contact onepress@plos.org.

You will receive an invoice from PLOS for your publication fee after your manuscript has reached the completed accept phase. If you receive an email requesting payment before acceptance or for any other service, this may be a phishing scheme. Learn how to identify phishing emails and protect your accounts at https://explore.plos.org/phishing.

If we can help with anything else, please email us at customercare@plos.org.

Thank you for submitting your work to PLOS One and supporting open access.

Kind regards,

PLOS One Editorial Office Staff

on behalf of

Dr. Firas H Kobeissy

Academic Editor

PLOS One
